# Differences between macrovascular and microvascular functions in pregnant women with chronic hypertension or preeclampsia: new insights into maternal vascular health

**DOI:** 10.3389/fphys.2025.1536437

**Published:** 2025-02-19

**Authors:** Julyane N. S. Kaihara, Hellen Cristiane Grepi Okano, Eduardo Carvalho de Arruda Veiga, Gustavo Moleiro Tallarico, Carlos Alan Dias-Junior, Ricardo Carvalho Cavalli, Valeria Cristina Sandrim

**Affiliations:** ^1^ Department of Biophysics and Pharmacology, Institute of Biosciences, Sao Paulo State University (UNESP), Botucatu, Brazil; ^2^ Department of Gynecology and Obstetrics, Ribeirao Preto Medical School, University of Sao Paulo (USP), Ribeirao Preto, Brazil

**Keywords:** arterial stiffness, chronic hypertension in pregnancy, endothelial dysfunction, preeclampsia, pulse wave velocity, peripheral arterial tonometry

## Abstract

**Introduction:**

Hypertensive disorders of pregnancy, including chronic hypertension (CH) and preeclampsia (PE), stand as prominent global contributors to maternal and perinatal morbidity and mortality. Endothelial dysfunction plays a central role in the pathophysiology of these conditions. This dysfunction impacts blood flow and the regulation of vascular response, potentially leading to alterations in the remodeling of blood vessels. Nitric oxide bioavailability, a key regulator of vascular tone, is often diminished in endothelial dysfunction, with nitrite levels serving as a surrogate marker. Methods such as pulse wave velocity (PWV) and peripheral arterial tonometry provide valuable insights into vascular health in large and small vessels, respectively, in hypertensive pregnancies. Among these, peripheral arterial tonometry stands out as a less explored technique in research. This study aimed to evaluate potential alterations in the macrovascular arterial stiffness and the microvascular endothelial function among pregnant women diagnosed with CH or PE compared to healthy pregnant (HP) women. Additionally, we aimed to correlate these vascular parameters with demographic and clinical data.

**Methods:**

The study enrolled 24 HP women, 24 with CH during pregnancy, and 24 with PE who underwent evaluations of large-artery stiffness via PWV assessments and peripheral arterial tonometry via natural logarithm of the reactive hyperemia index (lnRHI) assessments.

**Results:**

Patients with CH and PE exhibited higher large-artery stiffness than HP, although the lnRHI values remained comparable across all groups. Furthermore, PWV values demonstrated a direct correlation or tendency toward a positive correlation with systolic and diastolic blood pressures (SBP and DBP) in all groups. However, PWV and nitrite concentrations were not correlated. Notably, microvascular function was positively correlated with SBP and DBP in PE, but not in CH or HP. The correlation between lnRHI and nitrite concentrations was observed in the PE group.

**Conclusion:**

Thus, our findings indicate that, while HDPs have demonstrated increased large-artery stiffness in comparison to HP, the microvasculature analyzed by peripheral arterial tonometry was similar among all three groups. Interestingly, the correlation patterns in the nitrite levels, blood pressure, and microvascular function differed in the PE and CH groups.

## 1 Introduction

Hypertensive disorders of pregnancy (HDPs), including chronic hypertension (CH) and preeclampsia (PE), stand as prominent contributors to maternal and perinatal morbidity and mortality on a global scale (American College of Obstetricians and Gynecologists ACOG, 2020). CH affects 1%–2% of pregnancies and stands as the primary risk factor for the development of PE (Panaitescu et al., 2017), among other maternal characteristics.

One of the key factors in the development of both PE and CH is endothelial dysfunction. In PE, abnormal placentation and imbalanced placental factors in the maternal circulation cause endothelial injury, leading to impaired endothelial function (Tomimatsu et al., 2019; Powe et al., 2011), which is evidenced by a reduced flow-mediated dilation (FMD) in the vasculature (Cockell and Poston, 1997; Weissgerber et al., 2016). As a result of a decrease in vasodilator factors and an increase in vasoconstrictors (Tomimatsu et al., 2019), endothelial dysfunction significantly impacts blood flow and regulation of vascular response. This dysfunction can result in alterations in the remodeling of blood vessels, leading to arterial stiffening, impaired arterial compliance, and/or contractility in response to changes in blood pressure (Safar, 2018), and increased pulse wave velocity (PWV) propagation through the aorta and large arteries (Chirinos et al., 2019). Therefore, arterial stiffness can contribute to end-organ damage (Chirinos et al., 2019) and has a recognized prognostic value in assessing cardiovascular disease risk (Boutouyrie et al., 2021).

Nitric oxide (NO) is synthesized by nitric oxide synthase (NOS) through the catalysis of L-arginine. In endothelial cells, NO mediates smooth muscle relaxation in blood vessels by activating cyclic guanosine monophosphate (cGMP), which lowers intracellular calcium levels (Lowe, 2000). Accordingly, a decrease in NO bioavailability is recognized as a contributing factor to endothelial dysfunction, a condition associated with various cardiovascular disorders. As NO is a gaseous molecule with a relatively short half-life in biological systems, direct quantification of NO is challenging. Nitrite, a stable metabolite of NO, serves as a reliable surrogate marker for evaluating NO bioavailability in plasma.

Endothelial dysfunction can be evaluated by analyzing the vascular tone subsequent to the induction of reactive hyperemia through the temporary occlusion of the blood vessel. When the occlusion is released, the shear stress induced by the blood flow stimulates the endothelium. If the endothelial function is preserved, its cells will increase the NO production, resulting in a relative augmentation in blood vessel diameter (vasodilation) (Korkmaz and Onalan, 2008). The ultrasound assessment of FMD in the brachial artery is considered the gold standard for evaluating large-artery endothelial function (Stoner et al., 2013). In this context, peripheral arterial tonometry emerges as a novel and semi-automated method for assessing endothelial health, particularly regarding small-diameter blood vessels. However, the utilization of this methodology in research has been explored to a minor extent.

There are significant advancements in evaluating endothelial dysfunction using peripheral arterial tonometry, and most studies employing this method have focused on the detection of endothelial dysfunction in various pathological conditions, including PE (Meeme et al., 2017; Yinon et al., 2006; Carty et al., 2012). However, an important gap remains in the literature, as these studies have largely overlooked the specific group of pregnant women with CH and have not simultaneously evaluated arterial stiffness in these populations. Consequently, our study stands out by providing new insights into the vascular health of pregnant women with CH, contributing to a more comprehensive understanding of vascular complications in CH during pregnancy compared to PE. Therefore, we aimed to investigate potential variations in peripheral vascular endothelium and large-artery stiffness in women with CH and PE in comparison to normotensive women, by means of PWV and reactive hyperemia index (RHI) measurements. Additionally, we analyzed the correlations between these vascular parameters with demographic and clinical data, including plasma nitrite concentrations.

## 2 Methods

### 2.1 Study population

This case-control study was approved by the Ethics Committee of the University Hospital, Ribeirao Preto Medical School, University of São Paulo, Brazil (HCFMRP-USP) under protocol number 1.974.303 on 21 March 2017. The research was conducted in accordance with the institutional guidelines and regulations for research involving human subjects and in alignment with the Declaration of Helsinki. Participants recruited voluntarily provided written informed consent during clinical attendance. In the case of pregnant women under 18, consent was obtained from their parents or legal guardians. The study population enrolled a total of 72 women. It included 24 women with CH and 24 PE between 26 and 42 weeks of gestation who were followed up at the high-risk pregnancy outpatient clinic of the HCFMRP-USP. Moreover, 24 healthy pregnant women (HP, without HDPs or other complications) who attended the Reference Center for Women’s Health of Ribeirao Preto (MATER) were also included. CH in pregnancy was defined as sustained hypertension (systolic blood pressure, SBP ≥140 mmHg and/or diastolic blood pressure, DBP ≥90 mmHg) diagnosed before pregnancy or before 20 weeks of gestation, or if hypertension persisted for more than 12 weeks *postpartum*, in the absence of gestational trophoblastic disease (Vidaeff et al., 2019; Brown et al., 2018). PE was defined as new-onset hypertension diagnosed after 20 weeks of gestation associated with proteinuria (qualitatively identified via dipstick reading of 2+) or other end-organ damage, such as thrombocytopenia, renal insufficiency, impaired liver function, pulmonary edema, and visual symptoms (American College of Obstetricians and Gynecologists ACOG, 2020). Clinical data analysis and laboratory tests were conducted for the diagnosis of CH and PE, while ultrasound analysis was performed to identify a single fetus and determine corrected gestational age. All HDPs patients were receiving anti-hypertensive medications, including methyldopa and nifedipine, either as monotherapy or in combination. Data on delivery and newborn information were collected from medical records. Participants with multiple pregnancies or a history of fetal distress diagnosis, as well as those with positive serology for diseases such as human immunodeficiency virus (HIV), hepatitis B (HBsAg), hepatitis C (HCV), syphilis (VDRL), toxoplasmosis, or rubella, were excluded from the study. Additionally, participants with positive parasitological stool tests or urine cultures, type 1 diabetes, liver, heart, or kidney diseases that affect blood pressure, superimposed PE (when a CH patient develops any of the end-organ dysfunctions after 20 weeks of gestation consistent with PE) (Vidaeff et al., 2019; Brown et al., 2018), abnormal fetal ultrasound findings, those who developed hemolysis, elevated liver enzymes, and low platelet count (HELLP) syndrome, or those who withdrew during large-artery stiffness and peripheral arterial tonometry examinations were also excluded.

### 2.2 Large-artery stiffness assessment

Large-artery stiffness was evaluated using PWV measurement, which is a non-invasive method and the gold standard for assessing the stiffness of central muscular arteries. PWV data were recorded and analyzed via Sphygmocor Software version 9.0 (AtCor Medical, Sydney, Australia). Following a 5 min rest in an air-conditioned room, the participant was placed in a supine position. The external transducer was then placed directly on the skin over the right carotid artery and right femoral artery, and pulse wave velocities were recorded for a minimum of 10 s. The PWV was automatically computed as the ratio of carotid-femoral distance and the time interval between the two pulses (Hale et al., 2013).

### 2.3 Peripheral arterial tonometry

Endothelial function was assessed using the EndoPAT 2000 device (Itamar Medical Ltd., Caesarea, Israel) at the time of diagnosis, following the manufacturer’s instructions. This pneumatic plethysmograph identifies variations in the digital pulse waveforms known as peripheral arterial tone (PAT) signals by positioning probes on the distal phalanx of the fourth finger of both arms. The patients were placed in an air-conditioned room with a neutral temperature for the examinations. All objects that could interfere with the examination, such as restrictive clothing, jewelry, and accessories, were removed. Patients were made comfortable and allowed to sit or lie down in a relaxed position for a minimum of 15 min to ensure cardiovascular stability and acclimation to room temperature. Initially, a standard blood pressure cuff on the brachial artery of the non-dominant arm is set to induce a 5 min blood flow occlusion (Kuvin et al., 2003). Upon cuff release, blood flow increases (hyperemia), leading to endothelial-dependent flow-mediated vasodilation. The device detects this reactive hyperemia as an increase in the amplitude of the PAT signal. Subsequently, the device’s software calculates the RHI by comparing the ratio of the post- and pre-occlusion PAT signal in the occluded arm to that in the control (dominant) arm. The natural logarithm of the RHI (lnRHI) after vascular occlusion was used as it closely approximates a Gaussian distribution, with lnRHI ≤0.51 indicating abnormal microvascular function.

### 2.4 Plasma nitrite assay

The plasma nitrite concentrations were quantified using the Griess Reagent System (#G2930; Promega Corporation, Madison, United States), following the manufacturer’s instructions. Briefly, this assay involves incubations with sulfanilamide solution (1% sulfanilamide in 5% phosphoric acid) and NED solution (0.1% N-1-naphthyl ethylenediamine dihydrochloride in water) at room temperature. The absorbance of the colored compound formed by the reaction was then measured. Nitrite concentrations were determined by comparing the absorbance to a sodium nitrite standard reference curve.

### 2.5 Statistical analysis

The demographic and clinical variables as well as the PWV and lnRHI values of the subjects enrolled in this study underwent normality tests. For qualitative variables, One-Way ANOVA, Welch’s ANOVA, or Kruskal–Wallis test, followed by *post hoc* tests such as Tukey’s, Dunnett’s, or Dunn’s were applied as appropriate. The statistical analysis of categorical variables was conducted using Chi-square (χ^2^) or Fisher’s exact tests. A *p*-value ≤0.05 was considered significant. Pearson’s and Spearman’s correlation tests were performed to measure the linear relationships between the PWV and lnRHI data with the clinical characteristics of the subjects. A *p*-value <0.05 was considered significant for the correlations. All analyses were conducted via GraphPad Prism version 9.5 (GraphPad Software Inc., San Diego, United States).

## 3 Results

Table 1 provides an overview of the demographic and clinical characteristics of the 72 pregnant women included in this study. The HP and PE groups had similar ages, while CH patients were older than both. At the time of enrollment, CH and PE patients had higher body mass indexes (BMI) than HP women, and CH patients presented higher BMI values than PE patients. As expected, CH and PE patients presented higher SBP and DBP in comparison to the HP group, and the PE group also exhibited higher DBP than the CH group, despite both groups receiving anti-hypertensive therapy. Resting heart rates were similar across all groups. A comparison of the gestational age at sampling (GAS) revealed a similarity between the groups. However, the PE group exhibited an earlier GAS compared to the CH group. In terms of gestational age at delivery (GAD) and newborn weight, PE women delivered earlier and gave birth to smaller newborns compared to those in the HP and CH groups, as anticipated. The women with CH also delivered earlier than HP. Furthermore, newborns of PE women had a higher incidence of APGAR scores below 7 at the 1 min assessment after delivery, but no significant differences were observed during the 5 min assessment. Plasma nitrite concentrations were similar across all groups and are presented in Supplementary Figure 1.

**TABLE 1:** Demographic and clinical characteristics of all the subjects enrolled in this study.

Parameter	HP	CH	PE	*p*-value
Age (years)	27.2 ± 6.0	32.7 ± 5.4*	28.5 ± 5.8^#^	0.0038
BMI (kg/m^2^)	27.8 ± 4.2	36.1 ± 4.2*	31.8 ± 5.0*^#^	<0.0001
SBP (mmHg)	111.1 ± 12.2	135.3 ± 21.7*	147.0 ± 18.6*	<0.0001
DBP (mmHg)	74.5 ± 8.1	84.6 ± 11.0*	95.8 ± 13.5*^#^	<0.0001
Heart Rate (bpm)	76.0 ± 13.0	82.2 ± 15.0	78.6 ± 13.5	0.3081
GAS (weeks)	35.3 ± 2.3	36.5 ± 3.1	33.8 ± 4.0^#^	0.0184
GAD (weeks)	40.0 [37.0–41.0]	38.0 [34.0–42.0]*	36.0 [26.0–40.0]*^#^	<0.0001
Newborn Weight (g)	3,355.0 ± 324.6	3,236.0 ± 528.1	2020.0 ± 933.4*^#^	<0.0001
APGAR Score 1 (1 min) < 7	1.0 (4.2)	0.0 (0.0)	7.0 (33.3)*^#^	0.0010
APGAR Score 2 (5 min) < 7	0.0 (0.0)	0.0 (0.0)	1.0 (4.8)	0.4281

BMI, body mass index; CH, chronic hypertension in pregnancy; DBP, diastolic blood pressure; GAD, gestational age at delivery; GAS, gestational age at sampling; HP, healthy pregnant; PE, preeclampsia; SBP, systolic blood pressure. Data are expressed as mean ± SD, median [minimum-maximum], or absolute number (% of total). *p < 0.05 versus HP, ^#^ *p* < 0.05 versus CH.

**FIGURE 1:**
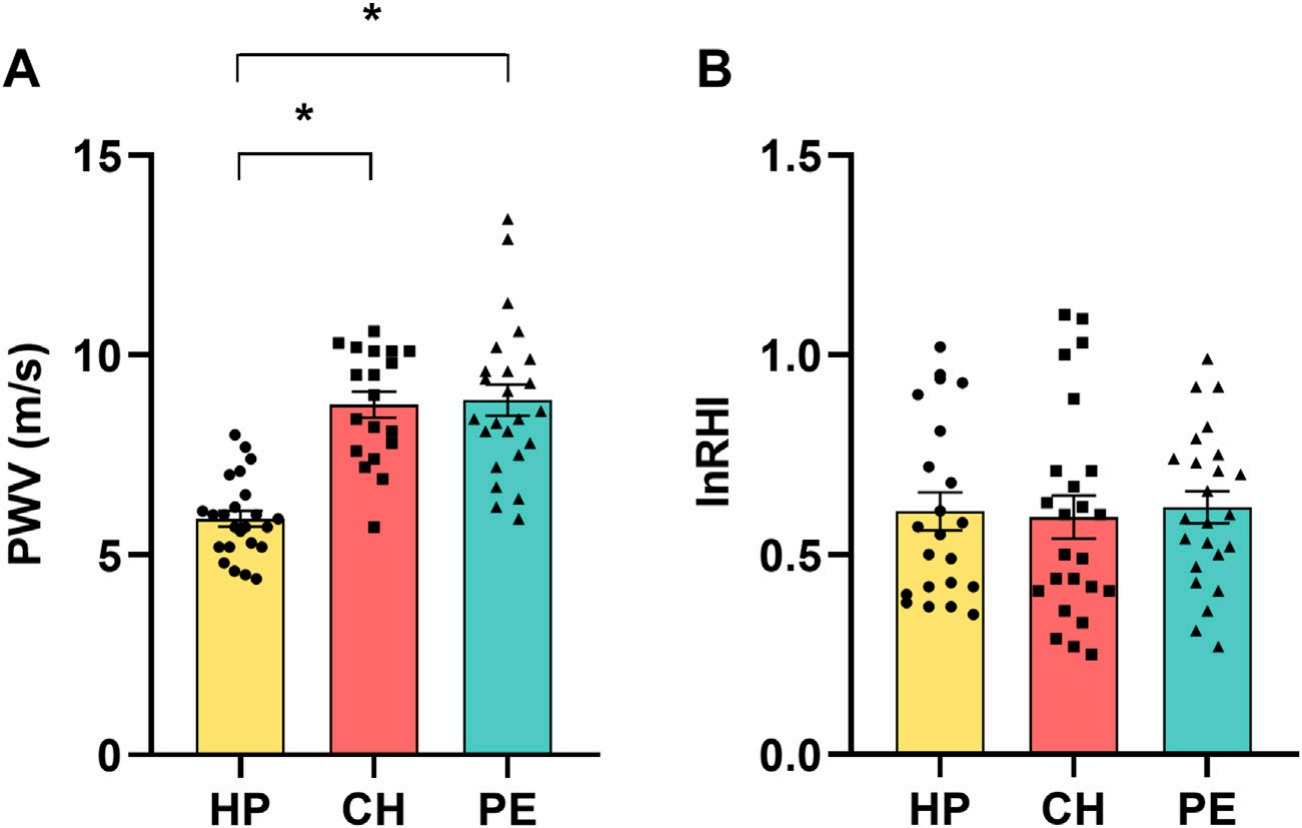
**(A)** Pulse wave velocity (PWV) and **(B)** natural logarithm of the reactive hyperemia index (lnRHI) in healthy pregnant women (HP), those with chronic hypertension during pregnancy (CH), and those with preeclampsia (PE). Data presented as mean ± SEM. *p ≤ 0.05 compared to HP.

Figure 1 displays the assessments of large-artery stiffness via PWV assessment (Figure 1A) and endothelial dysfunction in the microvasculature via peripheral arterial tonometry (Figure 1B). Compared to HP women, patients with CH and PE exhibited higher PWV values, indicative of large-arterial stiffness in those groups. The mean values of lnRHI among the HP, CH, and PE groups were similar. This suggests that the endothelial function in the microvasculature was comparable across all groups.

All patients enrolled in the CH and PE groups initiated anti-hypertensive therapy with pregnancy-specific medications following their respective diagnoses until delivery, including the day of sampling. Subsequently, a comparative analysis of PWV and lnRHI values was conducted based on the administered medication (methyldopa, nifedipine, or a combination of both). The results of this analysis are presented in Supplementary Figure 2. The findings revealed that both PWV and lnRHI demonstrated comparable outcomes across all subgroups, suggesting that these parameters remained unaffected by the anti-hypertensive medication.

**FIGURE 2:**
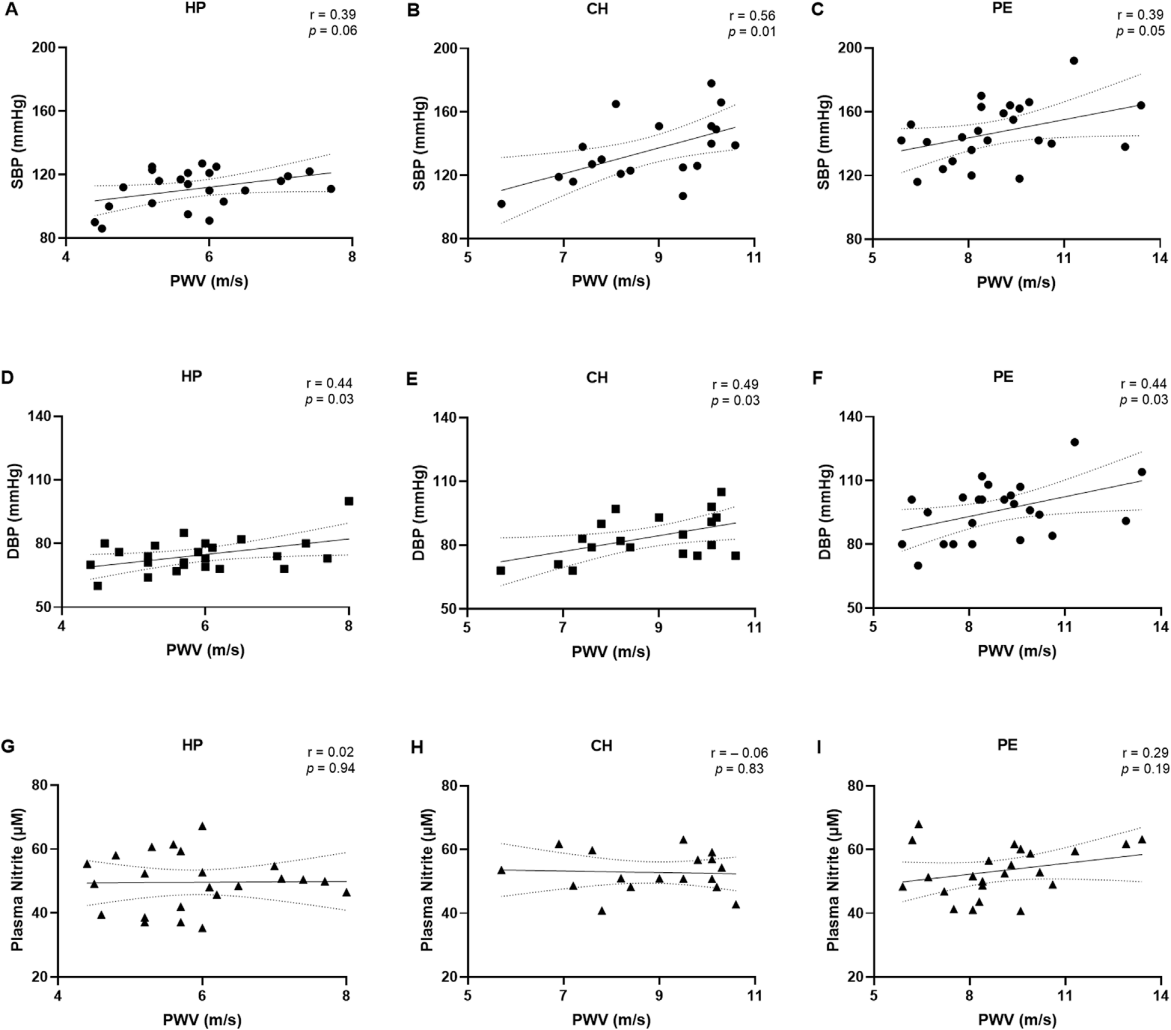
Correlations between pulse wave velocity (PWV) with systolic blood pressure **(A–C)**, diastolic blood pressure **(D–F)**, and plasma nitrite concentrations **(G–I)** in the healthy pregnant (HP), chronic hypertension during pregnancy (CH), and preeclampsia (PE) groups. Data presented as Pearson’s or Spearman’s correlation. r, Pearson’s or Spearman’s correlation coefficient.

Figure 2 and the Supplementary Table display the correlations between demographic and clinical data and PWV values. SBP tended to be positively correlated with PWV values in the HP (r = 0.39, Figure 2A), while this correlation was statistically significant in the CH group (r = 0.56, Figure 2B) and PE group (r = 0.39, Figure 2C). Similarly, DBP was positively correlated with PWV values in the HP group (r = 0.44, Figure 2D), CH group (r = 0.49, Figure 2E), and PE group (r = 0.44, Figure 2F). There was no correlation between PWV values and plasma nitrite concentrations among all groups (Figures 2G–I). Moreover, PWV values showed a tendency towards a positive correlation with BMI in the HP group, but no significant correlations were observed with other demographic or clinical parameters, including age, GAD, and newborn weight (Supplementary Table).

**FIGURE 3:**
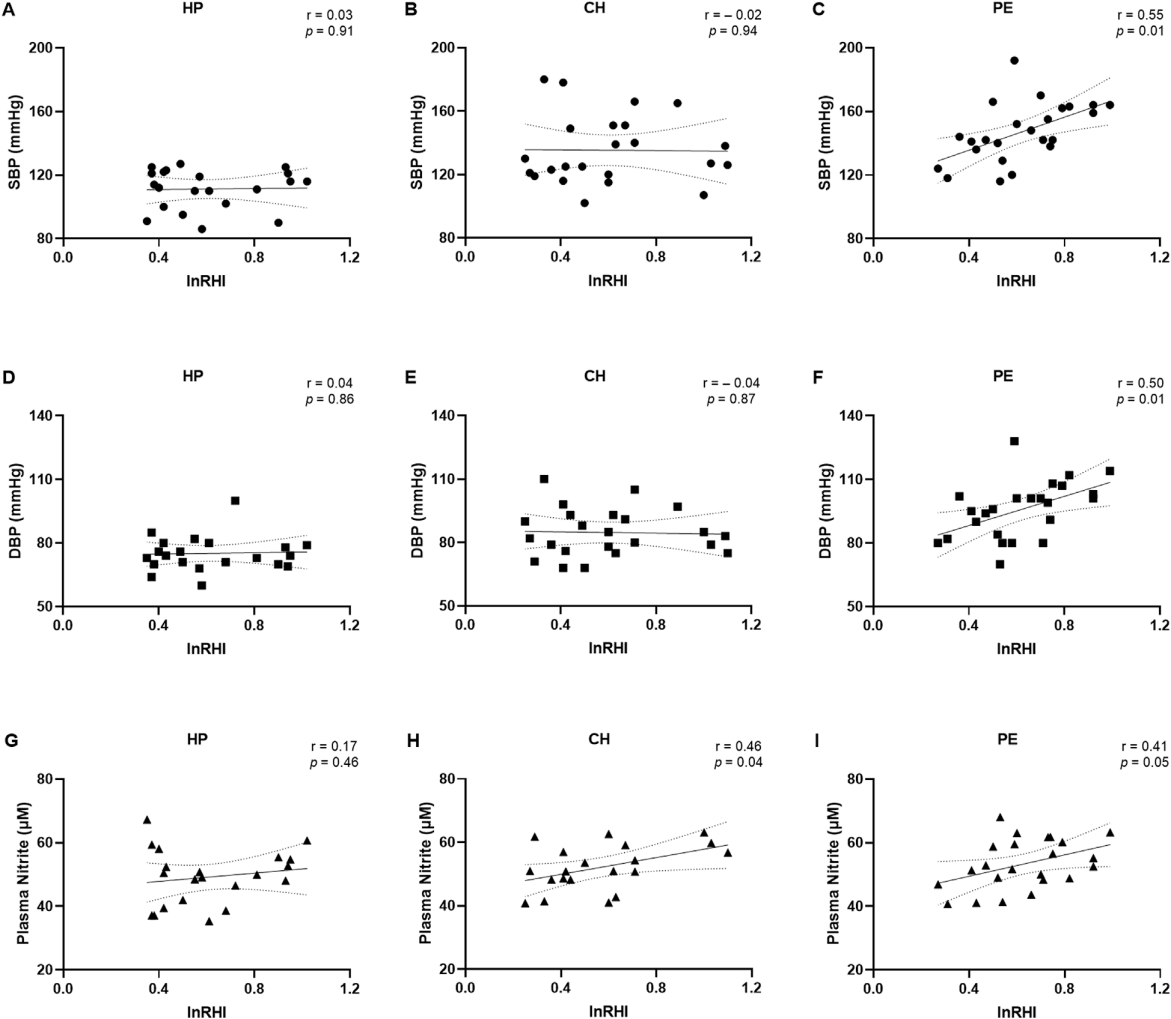
Correlations between the natural logarithm of the reactive hyperemia index (lnRHI) with systolic blood pressure **(A–C)**, diastolic blood pressure **(D–F)**, and plasma nitrite concentrations **(G–I)** in the healthy pregnant (HP), chronic hypertension during pregnancy (CH), and preeclampsia (PE) groups. Data presented as Pearson’s or Spearman’s correlation. r, Pearson’s or Spearman’s correlation coefficient.

The correlations between demographic and clinical data with the lnRHI values are presented in Figure 3 and the Supplementary Table. No correlations were observed when considering lnRHI with SBP and DBP in the HP (Figures 3A,B) or CH groups (Figures 3D,E). Nevertheless, in the PE group, a moderate positive correlation was observed between lnRHI with SBP (r = 0.55, Figure 3C) and between lnRHI with DBP (r = 0.50, Figure 3F), indicating that the higher the blood pressure, the higher the lnRHI. This is a notable finding, as greater values of lnRHI are associated with physiological endothelial-dependent vasodilation and normal endothelial function. In contrast to the findings observed in the macrovasculature, lnRHI was positively correlated with plasma nitrite concentrations in the CH group (r = 0.46, Figure 3H) and in the PE group (r = 0.41, Figure 3I). However, no such correlation was evident in the HP group (Figure 3G). It is worth noting that after excluding the three overt outliers in the correlation graph between lnRHI and plasma nitrite, the correlation became non-significant. There was no correlation between age, BMI, GAD, and newborn weight with lnRHI values across the three experimental groups (Supplementary Table), which suggests that the endothelial function may be independent of these demographic and clinical parameters in this study population.

**FIGURE 4:**
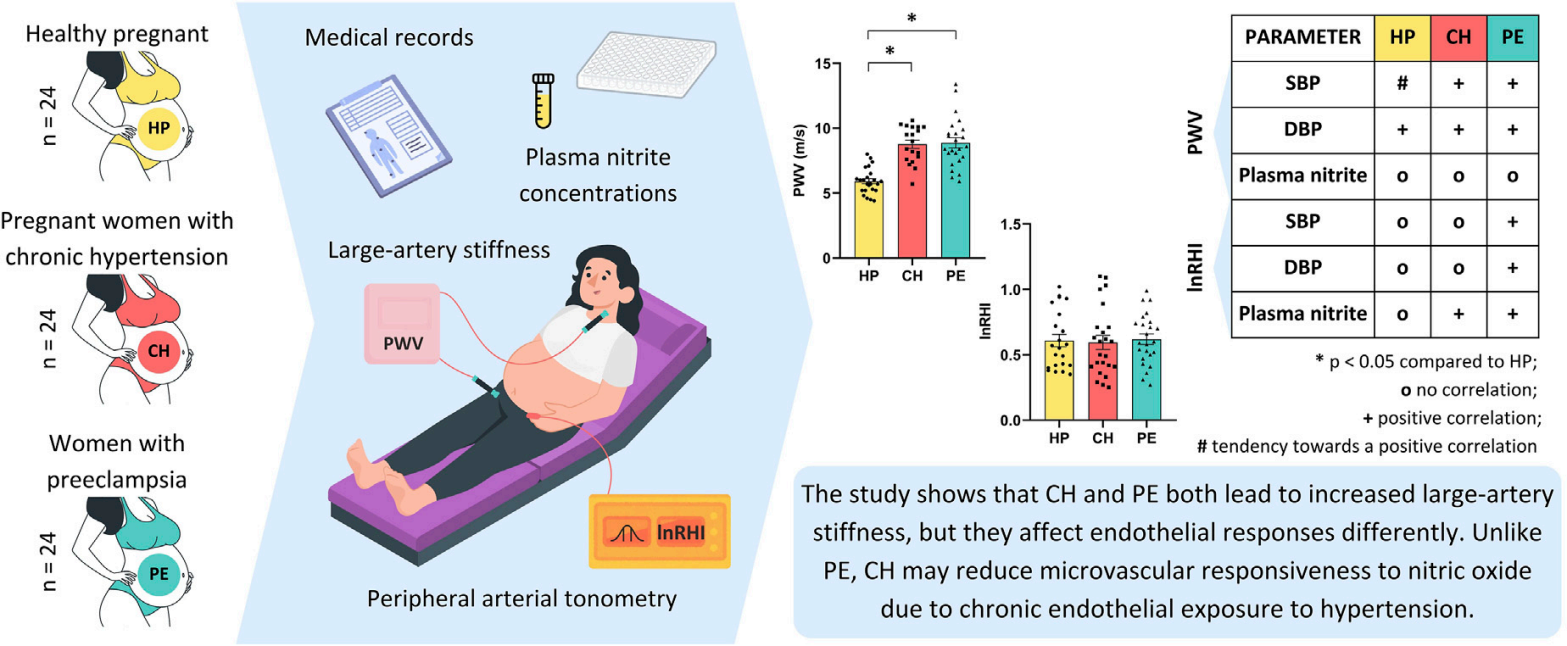
Graphical abstract.

Figure 4 presents an overview of the study design, methodologies, and key findings. This graphical abstract summarizes the analyses of macro- and microvascular functions in the HP, CH, and PE groups.

## 4 Discussion

In this study, we investigated the macrovascular arterial stiffness and microvascular endothelial function in the context of two HDPs–CH and PE–in comparison to normotensive HP women. Pregnant women with CH and PE showed significant large-artery stiffness compared to those with HP. In all groups, SBP showed a positive correlation or a tendency towards a positive correlation with arterial stiffness, with a similar association observed for DBP. Nevertheless, plasma nitrite concentrations were not correlated with arterial stiffness. Endothelial function in the microvasculature was comparable across all groups. However, our findings indicated that higher values of lnRHI in the PE group were associated with higher SBP and DBP, whereas no such correlations were observed between SBP and DBP in HP and CH. Additionally, there was a positive correlation between lnRHI and higher plasma nitrite concentrations in the CH and PE.

Our analysis revealed that large-artery stiffness was altered in the groups with HDPs, as evidenced by higher values of PWV. This finding is supported by other recent studies in other populations of patients with PE compared to healthy controls (Kaihura et al., 2009; Namugowa et al., 2017). Furthermore, women with a previous history of early-onset PE (Orabona et al., 2017a), and those with a previous history of PE with or without HELLP syndrome (Orabona et al., 2017b), continued to present altered arterial stiffness after delivery. Also, Hale et al. longitudinally evaluated the PWV of women during pre-pregnancy, early, and late pregnancy periods (Hale et al., 2013). The study found a difference in the pre-pregnant PWV between normotensive and hypertensive women, suggesting subclinical altered arterial stiffness in those with a predisposition to PE. Thus, our study not only provides additional information beyond the findings of Hale et al. (2013) indicating altered arterial stiffness in women with PE but also extends this understanding by revealing comparable alterations in women with CH in a larger cohort. The direct correlation between SBP, DBP, and PWV in the PE group may be attributable to the close association between arterial stiffness and its contribution to the development of hypertension. This association is further influenced by the high force of the blood against the artery walls, which leads to increased collagen production and elastin degradation (Mitchell, 2014) and influences the premature aging of the blood vessels (Shimokawa, 1998).

The physiological shear stress created by the blood flow over the endothelial cells activates mechanoreceptors and leads to a cascade of intracellular signaling pathways that result in the production of vasodilatory and growth factors to help maintain vascular tone (Davies, 2009). However, in hypertensive disorders, the impaired blood flow leads to abnormal shear stress, disrupting the hemodynamics and contributing to endothelial dysfunction by promoting a proinflammatory and prothrombotic state that further exacerbates vascular injury and stiffness (Davies, 2009). Our findings indicated that the endothelial state of women with HDPs and those with HP as assessed through peripheral arterial tonometry were similar. Limited research has been conducted on this topic, and previous reports have yielded conflicting results regarding the RHI of PE patients, even when using the same equipment. Differences in recruitment methodology relative to inclusion and exclusion criteria, or differences in the preparatory steps for the examinations could be reasons for this discrepancy. Yinon et al. reported that women with PE (n = 17 and mean GAS = 32.0 ± 4.0 weeks) exhibited lower RHI values than normotensive controls (Yinon et al., 2006). Similarly, Meeme et al. obtained the same result in a larger cohort (case group: n = 105 and mean GAS = 30.8 ± 0.4 weeks), but it is worth noting that it included HIV-positive patients (Meeme et al., 2017). When classified into subgroups of HIV-positive and HIV-negative patients, the difference in RHI between normotensive HIV-negative women and those preeclamptic HIV-negative lost significance, which is consistent with our findings.

Furthermore, the moderate positive correlation found between SBP and DBP with lnRHI in the PE group was noteworthy, as higher lnRHI values indicated a more responsive microvascular endothelial state. This may be attributed to the direct correlation between plasma nitrite and lnRHI, as well as the endothelial response to shear stress following cuff release, which stimulates the NO production in the microvasculature. Previous reports have indicated that the circulating nitrite levels were either increased (Noorbakhsh et al., 2013; Bartha et al., 1999) in PE or similar (Acauan Filho et al., 2016; Elmas et al., 2016) compared to healthy controls. Additionally, it has been demonstrated that serum nitrite levels were decreased in CH women in comparison to healthy controls (Bartha et al., 1999). As a response to the hypertensive state, the elevated nitrite levels, a biomarker of NO, observed in the PE group may indicate that NO plays a role in the response of the microvascular endothelium in PE, improving endothelial function. However, this does not appear to be the case in macrovasculature, as evidenced by the lack of correlation between PWV and plasma nitrite concentrations. Conversely, the same correlation of lnRHI with SBP and DBP was not observed in the CH group. This may be caused by established vascular structural and epigenetic remodeling (Davies, 2009; Mengozzi et al., 2023) as a result of the chronic changes in the hemodynamic state in these hypertensive patients, which impairs the endothelial response to shear stress. Therefore, these findings suggest that the differential correlation patterns between blood pressure and lnRHI in PE and CH are associated with the chronic nature of hypertension in CH. The blood pressure of this group was consistently elevated prior to the pregnancy. In contrast, the endothelial alterations observed in PE are less pronounced due to the new onset of hypertension during pregnancy, allowing for peripheral vasodilation. Further investigation is necessary to ascertain this hypothesis, as, to the best of our knowledge, there are no studies regarding the impact of the altered shear stress in the systemic vessels in conditions such as CH in pregnancy and PE.

This study has limitations. As this was a non-longitudinal study, our results elucidated associations between the variables under investigation and it was not possible to identify causal relationships, as the large-artery stiffness and peripheral endothelial function of the patients before and after pregnancy were unexplored. The observed discrepancies in BMI measurements among the study groups may be due to the inherent association between higher BMI values and an increased risk for CH and PE development. Although previous studies have shown a relationship between aging and vascular changes (Reference Values for Arterial Stiffness Collaboration, 2010; Mitchell et al., 2010), the age range in our study was relatively narrow. While we observed statistical differences in age between our groups, these age ranges are smaller compared to those evaluated in previous studies, which often include a broader spectrum of ages. Furthermore, our study revealed no correlation between endothelial function and age, or between arterial stiffness and age. Therefore, the impact of age on vascular changes in our study may be limited. Moreover, other biomarkers of vascular health that would provide a more complete understanding of vascular changes were not evaluated.

## 5 Conclusion

Our findings indicate that, while HDPs have demonstrated increased large-artery stiffness in comparison to HP, the microvasculature analyzed by peripheral arterial tonometry was similar among all three groups. Interestingly, the correlation patterns in the nitrite levels, blood pressure, and microvascular function differed in the PE and CH groups. These results bring new perspectives on endothelial function in macro- and microvasculature in HDPs and open new possibilities for diagnoses and treatments.

## Data Availability

The original contributions presented in the study are included in the article/supplementary material, further inquiries can be directed to the corresponding authors.
