# Supplementary material for: Differences between macrovascular and microvascular functions in pregnant women with chronic hypertension or preeclampsia: new insights into maternal vascular health
Source: Front Physiol. 2025 Feb 19;16:1536437. doi: 10.3389/fphys.2025.1536437 (PMC11880939; doi:10.3389/fphys.2025.1536437)
Supplement: Supplementary file 1 [file Table1.docx]

Supplementary Material

# Supplementary Figures


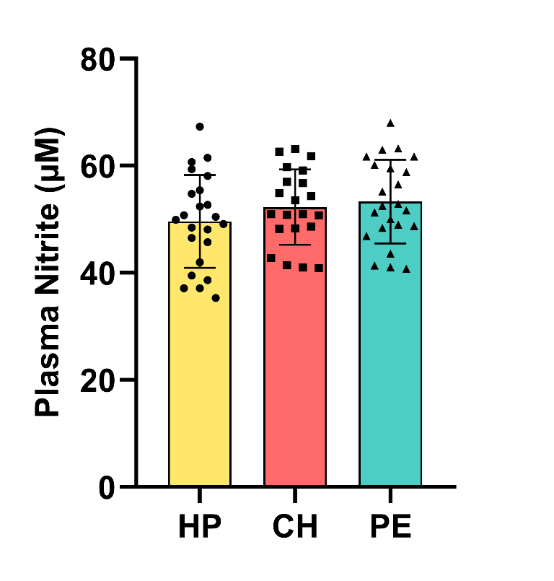


**Supplementary Figure 1.** Plasma nitrite concentrations in healthy pregnant women (HP), those with chronic hypertension (CH), and those with preeclampsia (PE). Data presented as mean ± SEM.

**
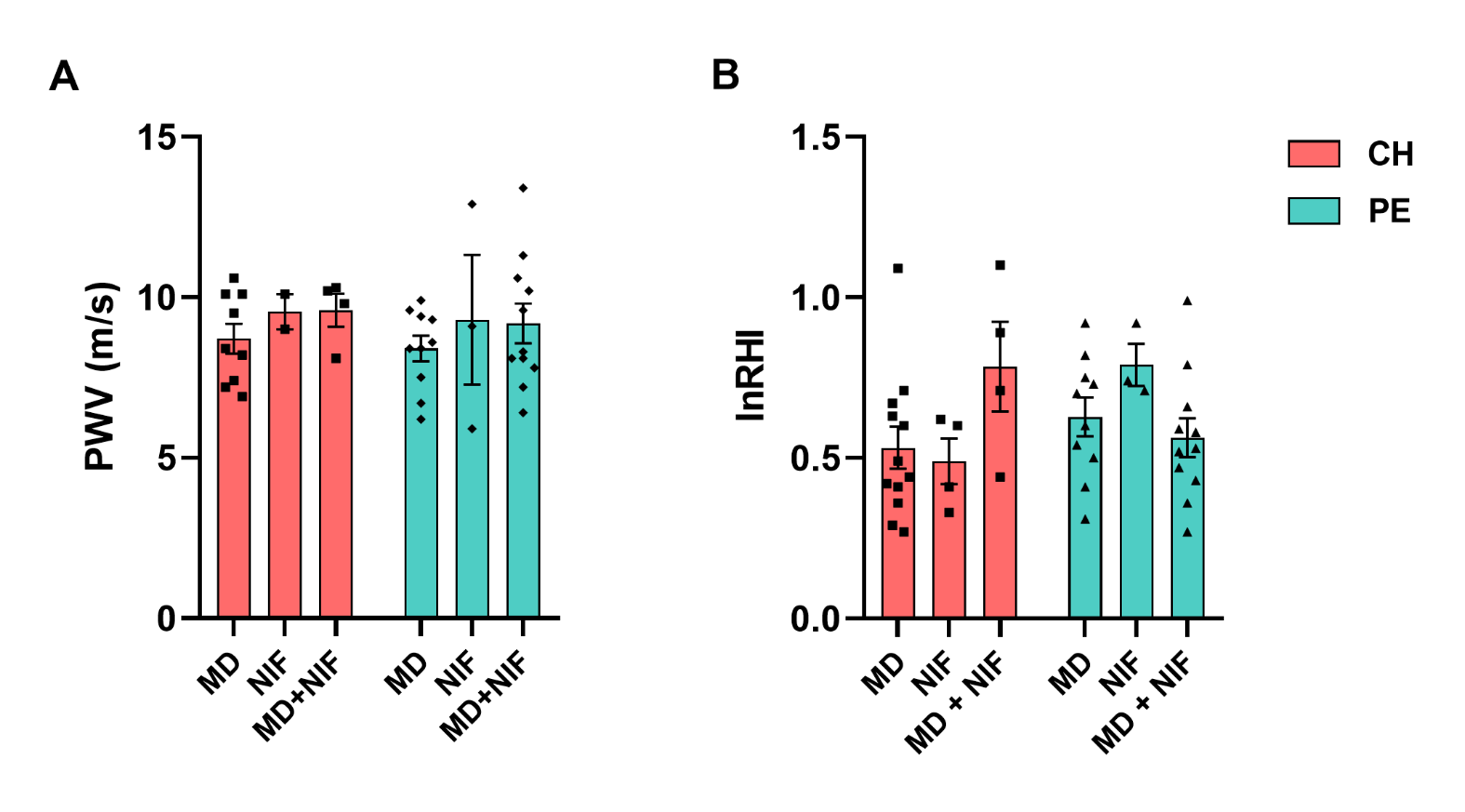
**

**Supplementary Figure 2.** (**A**) Pulse wave velocity (PWV) values of pregnant women with CH taking methyldopa (MD, n = 9), nifedipine (NIF, n = 2), or methyldopa + nifedipine (MD + NIF, n = 4); PE patients taking MD (n = 10), NIF (n = 3), or MD + NIF (n = 11) and (**B**) natural logarithm of the reactive hyperemia index (lnRHI) values of pregnant women with CH taking MD (n = 12), NIF (n = 4), or MD + NIF (n = 4); PE patients taking MD (n = 10), NIF (n = 3), or MD + NIF (n = 11). Data presented as mean ± SEM.

# Supplementary Table

**Supplementary Table.** Correlations between pulse wave velocity (PWV), natural logarithm of the reactive hyperemia index (lnRHI), and the demographic and clinical characteristics of all the subjects enrolled in this study.

| Parameter | HP | CH | PE |
| --- | --- | --- | --- |
| **PWV** | | | |
| Age (years) | r = 0.31, *p* = 0.15 | r = 0.16, *p* = 0.51 | r = 0.31, *p* = 0.14 |
| BMI (kg/m^2^) | r = 0.38, *p* = 0.06 | r = 0.26, *p* = 0.29 | r = 0.33, *p* = 0.13 |
| GAS (weeks) | r = – 0.01, *p* = 0.98 | r = 0.16, *p* = 0.58 | r = – 0.13, *p* = 0.54 |
| GAD (weeks) | r = – 0.06, *p* = 0.78 | r = 0.12, *p* = 0.63 | r = – 0.21, *p* = 0.33 |
| Newborn Weight (g) | r = – 0.20, *p* = 0.34 | r = – 0.01, *p* = 0.97 | r = – 0.02, *p* = 0.93 |
| **lnRHI** | | | |
| Age (years) | r = 0.23, *p* = 0.29 | r = 0.19, *p* = 0.38 | r = – 0.27, *p* = 0.20 |
| BMI (kg/m^2^) | r = – 0.03, *p* = 0.88 | r = 0.22, *p* = 0.31 | r = 0.17, *p* = 0.44 |
| GAS (weeks) | r = 0.06, *p* = 0.79 | r = 0.13, *p* = 0.58 | r = – 0.23, *p* = 0.27 |
| GAD (weeks) | r = – 0.19, *p* = 0.40 | r = 0.06, *p* = 0.80 | r = – 0.25, *p* = 0.25 |
| Newborn Weight (g) | r = – 0.04, *p* = 0.88 | r = – 0.21, *p* = 0.34 | r = – 0.17, *p* = 0.44 |

BMI, body mass index; CH, chronic hypertension in pregnancy; GAD, gestational age at delivery; GAS, gestational age at sampling; HP, healthy pregnant; PE, preeclampsia. r, Pearson’s or Spearman’s correlation.
